# Australian bus drivers’ modifiable and contextual risk factors for chronic disease: A workplace study

**DOI:** 10.1371/journal.pone.0255225

**Published:** 2021-07-29

**Authors:** Alison Brodie, Toby Pavey, Cameron Newton, Marguerite C. Sendall

**Affiliations:** 1 School of Public Health and Social Work, Faculty of Health, Queensland University of Technology, Brisbane, Queensland, Australia; 2 School of Exercise and Nutrition Sciences, Faculty of Health, Queensland University of Technology, Brisbane, Queensland, Australia; 3 School of Management, QUT Business School, Faculty of Business and Law, Queensland University of Technology, Brisbane, Queensland, Australia; Universitat de Valencia, SPAIN

## Abstract

**Introduction:**

Little is known about workplace health promotion for bus drivers. Bus drivers are at-risk of chronic disease because they are exposed to the risk factor combination of poor nutrition, low levels of physical activity, high levels of sedentary time and are often overweight or obese. The purpose of this paper is to situate the quantitative baseline data collected from bus drivers within qualitative findings about the socio-cultural context of the workplace.

**Materials and methods:**

Baseline data about physical activity, dietary intake and sedentary hours was collected from 58 drivers employed by a large bus company in southeast Queensland. Ten drivers and seven key workplace informants participated in interviews and discussion groups about driver health behaviours, workplace structure, culture, and resources.

**Results:**

The quantitative results of our study reveal bus drivers have a cluster of poor health behaviours—limited physical activity, lower than recommended consumption of fruit and vegetables and high levels of sitting time during working-hours—which contribute to a high prevalence of overweight and obesity and a heightened risk of chronic disease. The qualitative findings suggest there are individual and structural barriers to improving drivers’ modifiable health behaviours. Individual barriers include ingrained poor habits and more pressing life concerns, while structural barriers in the context of the workplace include time constraints, shift work, long days, a lack of work amenities and a general disconnect of drivers with their workplace.

**Conclusion:**

In this workplace, health promotion strategies for bus drivers should be prioritised as a means of improving drivers’ health. To maximise uptake and effectiveness, these strategies should make use of existing workplace resources and consider the context of workplace health behaviour change. Further research is warranted in a broader sample of Australian bus companies to explore the context of workplace health behavior change so targeted strategies to improve bus drivers’ health can be developed.

## Introduction

The 1953 seminal work by Morris and colleagues [1] highlighted sedentary London bus drivers were at greater risk of chronic disease compared to their more active bus conductor colleagues. Fast forward 65 years and bus drivers are still an at-risk group for chronic diseases. This isbecause the majority of this population are older, have a large waist circumference and are overweight or obese [2], sit for prolonged periods during driving and non-driving periods at work [2] during workdays, sit more than three hours longer than office workers [3, 4], and their work environment and lifestyle include unhealthy nutritional choices and minimal physical activity [2]. In particular, the risk factor combination of poor nutrition, low levels of physical activity and high levels of sedentary time, plus obesity, put this group at high risk of cardiovascular co-morbidities, with hyperglycaemia reported in 48% of this population [5]. Bus drivers are at higher risk of hypertension, diabetes mellitus, and dyslipidemia than general workers or propensity score matched controls [6] and have a higher prevalence of risk factors contributing to metabolic syndrome [7] and cardiovascular disease [6, 8, 9]. A similar chronic disease risk factor profile is shared by other professional truck and passenger transport drivers, who have a sedentary work environment, demanding work schedules, and limited opportunities for physical activity [10–20].

Like other professional drivers, bus drivers are considered a ‘hard-to reach’ population for workplace health promotion because most of their time at work is spent in a vehicle [5]. Given this population is highly likely to develop risk factors for cardiovascular disease, intervention strategies need to be developed and evaluated to promote physical activity and healthful eating behaviours and prevent excess weight gain. Previous research demonstrates workplace health promotion tailored to the mindset and health literacy skills of professional drivers can improve their health knowledge and cardiovascular health indicators [5, 21–25]. Similar positive results may be obtained with bus drivers—an under-researched group—where there is currently limited evidence regarding successful strategies to address lifestyle risk modification [18, 26].

The road transport industry (which includes truck, bus, tram and ferry service providers) is identified in the Australian Work Health and Safety Strategy 2012–2022 as a national priority industry for risk prevention activities, due to the high number and rates of work-related fatalities, injuries and illnesses [27]. Professional drivers are recognised in the Strategy as being highly prone to vascular diseases such as stroke, coronary artery disease, hypertension and heart failure as a result of their increased levels of obesity, smoking, sleep apnoea and low levels of exercise [28]. They are predisposed to disorders of the musculoskeletal system caused by the frequency and duration of time they spend seated [28]. Despite this, chronic disease risk prevention is not a key focus of the national strategy, which centres on reducing fatalities and injuries from vehicle incidents, assaults, acute muscular stresses, and falls [28].

Currently there is no other mandated legislation in Australia around chronic disease risk factor management in bus drivers. General guidelines and tool kits are available to help organisations develop their work processes to improve productivity and reduce chronic disease risks in their workers [29], however these are not specific to the road transport industry. In the absence of a dedicated wellness representative, bus companies may lack the time, knowledge or resources to access these guidelines and translate them into policy and practice in their workplace.

To add further, the Australian road transport industry, and the bus industry in particular, lacks baseline prevalence rates of driver health issues, as well as information on predictors of driver illness and injury beyond basic demographic and occupational information [17]. Further work is needed to characterise drivers’ work environment and lifestyle practices (particularly sleep, smoking, diet and exercise) and the social, environmental and regulatory contexts in which these exist. Various risk factor interactions may be influencing drivers’ health outcomes. These need to be understood so targeted and engaging health promotion strategies for bus drivers can be developed [18].

The use of emerging digital technology may be an effective strategy for reaching and engaging the bus driver population for health promotion [30]. Digital and mobile technologies are becoming a more prevalent means of communication in the transport industry [31, 32]. Of particular relevance to the time-pressured road transport industry, the burgeoning availability of smartphone technology and wireless internet access means digital technologies such as apps can be easily integrated into tools already used by the workplace and adapted to workers’ current state and context [33, 34]. To date, however, there has been little—if any—preliminary data about bus drivers’ health risk behaviours and work context for the purpose of developing digital or other health promotion strategies [18].

The aim of this research is to contribute knowledge about the prevalence of specific modifiable chronic disease risk behaviours in a sample of Australian bus drivers and to explore the context of these behaviours in the workplace. This knowledge will inform targeted workplace health promotion interventions using digital technologies for bus drivers. Physical activity, sedentariness and nutrition are the main focus of this research as these factors are known to impact chronic disease risk in other professional drivers [13, 16–18, 21]. Workplace health interventions targeting these factors can positively impact drivers’ health choices, prevalence of chronic disease, work performance and productivity [21, 22, 35, 36]. This paper reports quantitative and qualitative baseline findings as part of our ongoing multi-strategy Participatory Action Research (PAR) approach to inform, design and test effective health promotion interventions for bus drivers.

## Materials and methods

A bus company in south-east Queensland, Australia, was recruited into the project. Baseline data, including physical activity, dietary intake and sedentary hours, was collected from a volunteer sample of bus drivers to assist with the development of contextual interventions. Drivers and key workplace informants (depot, health and safety, and human resource managers) provided information about their work activities and workplace structure, culture, and resources in relation to driver health behavioursthrough interviews and discussion groups.

### Recruitment

Potential workplace recruits were identified via an internet search of bus companies located in south-east Queensland. The search focused on workplaces which expressed an ethos of staff health and wellbeing on their website. A web-crawler was used to obtain the email contact details of the Chief Executive Officer (CEO) or other high-level workplace manager, if these details were not available on the website. A letter and research team capability statement was sent directly to each CEO, explaining the purpose of our study, inviting their company to participate and providing the contact details of the research team. Ten bus company CEOs were approached. Approximately one week later, a reminder email was sent to the same 10 CEOs. Two CEOs responded, one of whom agreed for his company to participate in the study.

### Workplace description

The workplace is a large bus company (approximately 450 workers, of whom 387 (86%) are drivers) with four separate, individually operated depots based in south-east Queensland. In three depots, the company predominately operates school run and urban services while a rural depot operates long distance coach services between rural and regional areas. The mean age of drivers is 56 years (ranging from 20s to early 80s). According to data provided by the workplace, approximately 65% of drivers are full-time and approximately 30% are female. Most are day drivers working split shifts, for example, five hours in morning, four or five hours in evening with an interim non-work period.

The workplace has an ethos of caring for their drivers and has previously implemented workplace health promotion strategies. A low cost, community-based health and well-being program introduced in 2016, involving subsidised pool and gym membership, health presentations and free cholesterol checks, has not continued in its entirety. The main bus depot has a walking group, end-of-trip facilities (showers and change rooms) and a well-equipped kitchen. There are union-maintained vending machines in all depot kitchen areas although these do not have healthy options. All employees are invited to attend a free lunchtime barbeque at the end of each school term held at the main depot. The board is highly active in implementing strategies for fostering a positive and supportive organizational culture. Management attempts to motivate employees by championing positive health behavior and individual and collective achievement in physical activity/weight loss challenges through the ‘Hall of Fame’ noticeboards. The workplace has developed, and recently implemented, an integrated messaging app service for driver communication.

### Procedure

This research was approved by the Queensland University of Technology Human Research Ethics (approval number 1800000717). All participation was voluntaryAll workers provided their informed written consent prior to the commencement of data collection.

The research team had several informal meetings with key workplace informants, including the health and safety manager and depot manager at the main driver depot, prior to commencing data collection. This gave all parties an opportunity to discuss how the project might best address the needs of the workplace. Participatory Action Research methods were employed during the discussions via a circular process of data gathering and reflection. Key informants could engage with the project in a way specific to their workplace context and culture. Based on these informal discussions, the research team developed a draft action plan involving pre-intervention (baseline) data collection, intervention design and implementation and post-intervention data collection and evaluation. After baseline data collection, workplace managers were sent a summary of the key findings and a simple results infographic to share with their drivers. These formed the basis of further discussions between the workplace and the research team to plan intervention strategies targeted to the workplace need, context and culture. Draft action plans went through a circular process of revision and refinement prior to being finalised.

### Questionnaires

A self-completed questionnaire was modified from an instrument created for workers in the road transport industry and used by our group in a previous study [37]. The questionnaire included nutrition and physical activity questions from publicly available validated survey tools. The research team distributed project information sheets and questionnaires to drivers at the main depot at the end of their regular monthly drivers meeting. Drivers interested in completing the questionnaire provided their written consent and recorded their staff number on the questionnaire to protect their privacy. Drivers were informed their responses would be confidential. Completed questionnaires were collected by the research team. As not all drivers were able to attend the meetings, a key workplace informant distributed questionnaires to drivers as they saw them on an *ad hoc* basis over the week. Completed questionnaires were placed in a sealed box and returned to the research team via mail. Two of the depots were not able to provide a time at which a group of drivers would be available to complete questionnaires *en masse*. At these locations, a key informant circulated questionnaires and collected and returned completed questionnaires by mail.

Part 1 of the questionnaire included items about age, gender, number of hours worked on a typical day and over the previous four weeks, type of driving (long distance or day trips), self-rated overall health, source of health information, readiness to make lifestyle changes to improve health and self-reported height and weight.

Part 2 investigated intake of fruit, vegetables, unhealthy food and sugary drinks. Questions included: ‘How many serves of a) fruit, and b) vegetables (fresh, tinned, frozen) do you usually eat each day?’, ‘On how many days of the week do you usually eat unhealthy foods high in saturated fat, added salt or added sugar?’ and ‘How many cans of sugary drink do you usually consume each day?’

Part 3 measured physical activity, by asking ‘How many times a week do you usually do 30+ minutes of moderate intensity physical activity? (e.g. brisk walking, etc.)’, ‘How many times a week do you usually do 15+ minutes of vigorous intensity physical activity? (e.g. heavy loading, jogging, fast cycling, etc.)’ and ‘How many hours do you spend a) sitting (including driving), b) standing, c) walking, and d) doing heavy labour or physically demanding tasks, on a typical work day. Six questions asked about drivers’ environment at work in relation to physical activity: ‘Does your workplace provide education on physical activity?’, ‘Does your workplace have standards or policies for physical activity?’, ‘Physical activity is encouraged at my work’, ‘It is easy for me to be active during my work day’, ‘Other truck drivers at work are physically active’ (Likert scale from ‘strongly disagree’ to ‘strongly agree’) and finally,‘Who do you think should be most responsible for helping road transport workers to eat well and be physically active at work?’ (‘individuals’, ‘workplaces’ or ‘the transport industry’). The same set of questions was asked about drivers’ work environment in relation to healthy eating. Additional questionnaire items about the use of digital technologies and preference for interventions are not the focus of this paper.

### Driver discussion groups and manager interviews

Ten bus drivers volunteered to participate in small discussion groups to provide richer, more contextualised information about whether any situational (such as access to healthy foods or recreation areas), personal or cultural barriers or facilitators affected their ability to make healthy choices at work. Drivers were asked to describe their work activities, physical activity and food choices at work, barriers to physical activity and good nutrition at work, thoughts about how these issues could be addressed and the role the workplace plays/should play in supporting drivers to be physically active, sit less, and make healthier food choices (S1 Appendix). Additional discussion group and interview questions about use of digital technologies and preference for interventions are not the focus of this paper. Similar questions were posed to 7 workplace managers in separate small group interviews. The interviews and discussion groups were semi-structured, with questions adopted from our previous study of workers in the road transport industry [37]. The aim was to gather rich contextualized information specific to individual workplaces, rather than to gather data for replication of analyses.

All discussion group and interview participants were provided with a project information sheet prior to the interviews and were reassured about confidentiality. Participants were free to withdraw from interviews at any time. All interviews were conducted by the research team leader and were digitally recorded with the written consent of all participants.

### Data analysis

A research assistant entered questionnaire data into an Excel spreadsheet. Data from the four depots were pooled and tallied for each questionnaire variable. Selected descriptive statistics relating to respondents’ demographic characteristics, typical dietary intake, physical activity, and sedentary behaviour are presented in Table 1. Data from some variables was collapsed into categories to improve the brevity of the table.

**Table 1:** Selected characteristics of bus driver questionnaire respondents (n = 58).

Age (average)	52 years	
	N	(%)
**Sex**		
Male	39	67.2
Female	19	32.8
**Type of driving**^a^		
Local (day) trips only	50	90.9
Long distance (overnight) across state	2	3.6
A mixture of 2 or 3 of these	3	5.5
**Average number of hours worked on a typical day**^b^		
Up to 7	26	52.0
8 or 9	12	24.0
9+	12	24.0
**Self-reported health**		
Fair or Poor	12	20.7
Good	36	62.1
Very good or Excellent	10	17.2
**Readiness to make a health change**^c^		
Do not want or need to change	8	14.2
Planning to, or thinking about making changes	11	19.6
Have made and maintained changes	10	17.9
Currently making changes	27	48.2
**Body Mass Index (BMI)**^d^		
18.5–24.9 (Normal)	8	15.7
25–29.9 (Overweight)	13	25.5
30+ (Obese)	30	58.8
**Consume recommended 2 serves of fruit/day**^e^	25	44.0
**Consume recommended 5 serves of veges /day**^f^	6	11.0
**Days per week of unhealthy food**^g^		
0	9	15.8
1 to 2	31	54.4
3 to 4	11	19.3
5+	6	10.5
**Cans per day of sugary drinks**		
0	30	51.7
1 to 2	23	39.7
2 or 3	5	8.6
**Hours sitting at work each day**^h^		
3 to 5	8	15.7
5 to 7	13	25.5
7+	30	58.8
**Times per week of 30 minutes of moderate physical activity**		
0 times	15	25.9
1 or 2 times	17	29.3
3 or 4 times	20	34.5
5+ times	6	10.3
**Times per week of 30 minutes of vigorous physical activity**		
0 times	30	51.7
1 or 2 times	17	29.3
3 or 4 times	8	13.8
5+ times	3	5.2
**It is easy for me to be active during my work day**^i^		
Disagree or Strongly Disagree	20	35.1
Neutral	26	45.6
**It is easy for me to eat healthy foods during my work day**		
Disagree or Strongly Disagree	16	27.5
Neutral	17	29.3
Agree or Strongly Agree	25	43.1
**Who should be most responsible for drivers to eat well and be physically active?**^j^		
Individuals	40	80.0
Individuals and workplaces	2	4.0
Workplaces	2	4.0
The transport industry	6	12.0

^a-j^ Missing data: ^a^3,^b^8,^c^2,^d^7,^e^1,^f^1,^g^1,^h^7,^i^1,^j^8.

Respondents who reported eating two or more servings of fruit per day and five or more servings of vegetables per day, were considered to have met the recommended daily intakes of fruit and vegetables, respectively [38].

Body Mass Index (BMI) was calculated for each respondent using their self-reported height and weight according to the formula BMI = kg/m^2^ where kg is a person’s weight in kilograms and m^2^ is their height in metres squared. BMI values were collapsed into categories based on Australian standards [39].

Audio data from interviews and discussion groups were transcribed by a research assistant who was present during the interviews. Transcripts were analysed using an inductive approach and thematic analysis to identify themes within the data. In this paper, qualitative findings from interviews and discussion groups are summarised to position the quantitative data within the context of the workplace. More detailed analysis and qualitative findings will be explored in a future paper.

## Results

### Questionnaires

Fifty-eight driver respondents completed the questionnaire out of a total driver population of 387 across the four depots. Selected driver characteristics are shown in Table 1.

The mean age of the driver respondents is 52 years (range 33 to 82 years). Most respondents (90.9%) only do local day trips while a small proportion do long distance overnight trips (3.6%) or a mixture of local and overnight trips (5.2%). Approximately one third (33%, n = 19) of respondents are female. Just over half (52%) work in split shifts up to 7 hours a day, 16% work 8 hours a day, 8% work 9 hours a day and 24% work more than 9 hours a day.

Only 10.3% of respondents meet the Australian recommendations of 150 minutes of moderate-to-vigorous physical activity per week [40]. Just over a quarter (25.9%) of respondents do not do any 30-minute episodes of moderate intensity physical activity in an average week and 51.7% of respondents do not do any 30-minute episodes of vigorous intensity physical activity in an average week. Approximately 60% of respondents sit for 7 or more hours per day at work. Most respondents do not meet the national recommendations for fruit and vegetable consumption (44% of respondents consume the recommended 2 serves of fruit per day while only 11% consume the recommended 5 serves of vegetables). Just over half (54.4%) of respondents report they eat unhealthy food such as burgers, chips, pies or cake, on one or two days per week and almost half (48.3%) consume between one and three cans of sugary drink per day. Only 15.7% of respondents are in the normal healthy weight range (Body Mass Index [BMI] 18.5–24.9 kg/m^2^) with 25.5% of respondents being overweight (BMI 25–29.9 kg/m^2^) and 58.8% being obese (BMI 30+ kg/m^2^).

Just over three quarters (77.6%) of respondents self-report their health as being ‘good’, ‘very good’ or ‘excellent’. The majority report they are either ‘currently making changes’ (48.2%), ‘have made and maintained changes‘ (17.9%) or are ‘thinking about’ (12.5%) making changes to improve their health. Approximately 80% of respondents believe they should take personal responsibility for eating well and being physically active, with 12% believing the larger transport industry, and 4% believing the specific workplace should take responsibility for drivers’ health.

Respondents offer mixed views about whether it is easy for them to be physically active during their workday. Only 19.3% of respondents agree it is easy for them to be active during their workday while half (45.6%) are ‘neutral’ (neither agree nor disagree) and 35.1% ‘disagree’ or ‘strongly disagree’. When asked if their workplace encourages them to be physically active, 44% of respondents are ‘neutral’ while 19% ‘strongly disagree’ and 19% ‘agree’.

Approximately 43% of respondents ‘agree’ or ‘strongly agree’ it is easy for them to eat healthy food during their workday while 27.5% either ‘disagree’ or ‘strongly disagree’. Approximately half (45.6%) of respondents are ‘neutral’ (neither agree nor disagree) while almost equal proportions ‘strongly disagree’ (19.3%), ‘disagree’ (15.8%) or ‘agree’ (19.3%) healthy eating is encouraged at work.

### Driver interviews

During interviews, drivers identify several common issues influencing their level of physical activity, sedentary behaviour and dietary choices. Working schedules, including time pressures, split shifts and long days are a significant barrier. Sometimes drivers do split shifts where they work for 5 hours in the morning and 5 hours in the afternoon and within a timeframe of 15 hours. Drivers say the nature of the work means they are highly sedentary although some drivers on spilt shifts are physically active cleaning their bus in between shifts: “*So in between his two driving shifts he’s out cleaning*, *sweeping*, *washing*, *mopping constantly in and out moving*.” For most, the pressures of working to strict timetables leaves little time for any exercise: “*It’s hard outside of work to be able to be involved in sport or things that are on at a regular time because our shifts are all over the place”*. Meal breaks are often short and very irregular, meaning drivers often rely on highly processed convenience food which could be purchased and consumed quickly: *“It’s just easy to go and get lunch in the KFC*, *instead of wasting 10 minutes in a half hour break to get it down*.” Drivers doing some shifts are not given paid meal breaks.

As frequently noted, split shifts create long days. Drivers often leave for work shortly after waking and go straight to bed as soon as they return home in the evening and are too tired for any extra activity: *“If you feel drained*, *you don’t have the energy to actually do any physical*.” For some, preparation to get the bus road-ready consumes time before and after driving the bus and/or in the middle of the day between shifts. For these reasons drivers often skip a healthy breakfast or home-cooked dinner. Some drivers who work in split shifts for school runs work a second job in between morning and afternoon shifts at the bus company: “*I do 3 hours in the morning in driving and then I drive here cause I’ve got two jobs and then I do three hours of cleaning and then I go back and drive three hours*.” Not surprisingly, several drivers express a general disconnect with their workplace as they are rarely there and lack close communication with their managers and colleagues. Some drivers feel the workplace is only interested in keeping them on the run: “*The less people on the ground*, *the more they like it*.*”* This is particularly the case in urban depots which have a larger, more dispersed workforce and a wider variety of services provided for longer times.

Drivers find a lack of facilities and amenities to be another common barrier to physical activity and healthy eating. Drivers frequently mention they can’t keep pre-made healthy food on the bus or in the workplace as there are no refrigerated food or water storage facilities. A lack of exercise equipment and/or shower/change facilities means they can’t freshen up and look presentable (a requirement of the job) should an opportunity arise for exercise in between a split shift: “*I chose not to walk because I wouldn’t feel fresh when I get here*.” Some drivers specifically mentioned the heat as a big barrier to exercise: *“The only way that you could probably do walking is on the treadmills in the air con”*.

Despite the lack of facilities, drivers acknowledge the workplace tries to help them make healthier choices, however they do not feel it is the responsibility of the workplace to do so. Most drivers believe their health choices and motivation is their personal responsibility: “*It’s all up to you*.” Some say their poor physical activity and diet choices are tied up in their old bad habits which they find very difficult to break. Other drivers say they lack knowledge about healthy eating (food preparation and portion size) while others have the knowledge but lack the motivation or the financial ability to afford to eat more healthily. As expressed by one driver: *“The best thing the company can do is subsidise costs for drivers who want to help themselves”*.

### Manager discussion groups

Managers say their workplace culture is very supportive of their drivers’ health which they view as a “positive investment” and drivers are considered part of the family. The workplace has previously made attempts to provide information and develop a health program for their drivers. However a lack of funding and—except for a small group—driver apathy, stymied further developments. Managers describe drivers’ age (*“I’ll do what I wanna do at this age”*), established habits and resistance to change (*“by the time they’re 60 they’re set in their ways*, *it’s hard to change”*) as strong contributors to their lack of engagement with workplace health initiatives. The exception is if a particular driver or drivers experience their own health crisis which often makes them an advocate for healthy choices around physical activity and improved nutrition.

Managers readily acknowledge the nature of the work (split shifts and time pressure: “*I think part of the problem from a health and wellbeing perspectives is the split shifts*”, and lack of exercise facilities in the workplace makes it difficult for drivers to make healthy choices. They note a high driver reliance on convenience food and acknowledge the workplace contributes to this with junk food vending machines and a lack of food storage facilities and preparation facilities at work. Drivers who service some major urban bus interchanges have access to lunch facilities (refrigerator, microwave, cutlery) and these are appreciated and well-utilised. Similar facilities are available at the large and recently refurbished main bus depot but facilities are limited at other depots.

Managers acknowledge air conditioning, and gym facilities or treadmills at work would help drivers exercise, but structural and financial constraints and workplace health and safety issues around onsite exercise equipment present difficulties. This, and differing management and communication styles, as well as frequent management change at particular depots, are noted to impact negatively on drivers’ choices and health. Some managers in smaller depots have a close personal relationship with their drivers and are heavily invested in improving their health. Other managers express a difficulty in knowing how best to connect with their driver workforce and engage them in activities to improve their health: “*It’s the engagement that’s the issue”*.

## Discussion

The road transport industry is one of the highest risk industries for poor worker health and wellbeing, mostly because drivers’ health is compromised by the requirements and limitations of their job [2]. Bus drivers in particular are a poorly researched groupand there is little available information about health behaviours to guide workplace health promotion strategies. Here, we investigate the prevalence of health behaviours such as physical activity and diet which contribute to chronic disease risk in Australian bus drivers and explore the context of these behaviours. We found a cluster of poor health behaviours influenced by organisational and individual factors.

Our driver sample had a high prevalence of overweight (26%) and obesity (56%). International studies of bus drivers have found levels of overweight and obesity ranging from 41% overweight and 21.1% obese (Nigeria) [8] to 43.3% overweight and 22.2% obese (India) [41] to 53.9% combined overweight and obese (Korea) [42] to 56% (obesity only) (United States) [43] to 91.1% (combined overweight and obese) (United States) [44]. This makes drivers in our sample sit at the top end of obesity prevalence internationally (along with drivers from the United States) and sit just behind United States drivers when combining both obesity and overweight prevalence.

Similar to previous findings [44], most drivers in our study do not meet the national recommendations for physical activity. Approximately 10% of drivers do meet the recommendations and around a third just fall short. This variation may result from different types of driving and shifts. Drivers on split shifts often clean buses or work a second job between shifts, which would increase their physical activity compared with drivers doing charter work or long runs. The number of hours at work would influence time available for exercise, and this was variable in our driver sample.

Yeary et al. [44] found bus drivers who did not meet physical activity recommendations have a higher BMI (36.5 kg/m^2^) than drivers who met recommendations (30.9 kg/m^2^). We did not investigate this in our study. Others [45] found male bus drivers are more physically active during leisure time than work time but still have a significantly higher BMI than other men. We did not investigate leisure time physical activity but it is possible any increase in leisure time physical activity would not be sufficient to compensate for the obesogenic impact of sustained reduction in physical activity during workdays. It may be possible individuals with a high BMI including women (who made up 30% of our sample), are attracted to bus driving as a profession.

Most of our driver population report being sedentary for 7 or more hours per day. Other driver studies have found sedentary time to be significantly higher on workdays than non-workdays and during working-hours than non-working-hours [2]. By contrast, a study using objective measures of sedentary time in Australian bus drivers [46] found sedentary time to be significantly higher on leisure than work days. On workdays, sedentary time was significantly lower when drivers were working (44%) than when not working (60%). These results challenge the perception that a sedentary occupation such as bus driving necessitates an inactive work environment [46]. Again, the type of shift and bus runs may influence these findings.

In our sample, drivers’ dietary choices are largely influenced by a need for convenience and a lack of facilities for storing or preparing food at work. Consequently, consumption of fruit and vegetables is lower than recommended in national guidelines. Fast food is consumed by most drivers several times during the working week. We and others found healthy eating and weight management are generally perceived to be difficult at the workplace [43].

In discussions with drivers and managers, we found drivers’ ability to make positive changes to increase their physical activity and improve their diet is strongly impacted by organisational, structural and personal factors. The very nature of bus driving (largely sedentary, with tight deadlines, early starts and late finishes, and split shifts) stresses and fatigues drivers [47] and for drivers in certain shifts, presents little opportunity for incidental or weekday out-of-hours physical activity. This contributes to obesity. Rosso et al. [48] found high driving hours per day and distance travelled per year to be associated with obesity risk. In US bus drivers doing school runs, structural barriers, sedentary hours and work stress were significant barriers to achieving a healthy weight [44]. Simply being a bus driver has been shown to be a significant independent predictor of chronic heart disease [45].

We and others [44] found resources for physical activity and healthy eating are generally limited in bus depots, although this may vary workplace to workplace and depot to depot. This is highly impactful as if drivers are not driving their bus, they are generally in their depot. Similarly, organisational culture can vary between workplaces/depots and impact on the financial and managerial support given to driver wellness and workplace health promotion. Managers in our study expressed positive intentions but lacked the financial capacity to alter existing infrastructure to accommodate exercise or food preparation/storage facilities. In addition, managers expressed difficulties in communicating and engaging with drivers who are largely stressed and time poor and overloaded with messages and deadlines. These structural factors are challenging but not insurmountable and attest to the importance of utilising and tailoring existing workplace resources.

It is prudent to consider how the personal characteristics of bus drivers may impact their health behaviour. Most drivers are older (50+) and this may exacerbate degenerative musculoskeletal issues such as arthritis, osteoporosis and sarcopenia, which can cause pain, limit physical activity and increase risk of injury [49–52]. Many individuals come to bus driving later in life, commonly after driving professionally in other capacities, for example, truck driving. Risk factors such as overweight/obesity, poor diet and lack of physical activity may thus have been carried over from previous driving occupations. Even if not, individuals who are already overweight or obese may be drawn to bus driving as it is a largely sedentary occupation which their weight (within reason) does not preclude them from performing.

As mostly male, low-salaried blue-collar workers, bus drivers fall into several other at-risk categories including low educational attainment, poor health literacy and frequently, a culturally and linguistically diverse background [53–55]. Some drivers in this study lacked knowledge about healthy eating including how to choose, prepare and portion healthy food and how to shop to a budget. Some drivers said they could not afford the cost of fruit and vegetables and enjoyed their habitual consumption of convenience food. They expressed more pressing life concerns than their own physical activity and nutrition issues, which was not often spoken about in the largely traditional older male work culture. Female drivers participating in the discussions placed relatively more value on their health behaviours.

Of note, most drivers believe the choices they make around their health are their own personal responsibility. Many report they have recently made or are currently making changes to improve their health. The finding that drivers find it easier to eat healthy foods while at work than be more physically active at work is not surprising as drivers can make a choice about what they buy or bring to eat (in their limited time) whereas they cannot interrupt their bus runs to exercise. This may place more of an onus on the workplace, and less on the drivers themselves, to find novel strategies to increase physical activity. Encouragingly, others have found tailored workplace health promotion programs for bus drivers to be effective in increasing physical activity and other health behaviours [7, 56–58].

### Limitations

The findings of this study may be limited by several factors. We only report the results from a single bus company, self-selected to be interested in driver wellness. The small sample size (approximately 15% of drivers) means findings may not be representative of the larger driving workforce and there were not enough questionnaire respondents to look at differences between urban and regional depots. Our lack of objective measures of driver weight, height, physical activity, sedentary behaviour and dietary intake may mean our self-reported data is an over or under-estimate of actual measures. We did not investigate leisure versus work health behaviour. Our findings, however, provide one of the first insights into the physical activity and diet behaviours of Australian bus drivers and the factors which impact upon them, and serve as a basis for future workplace interventions to improve drivers’ health.

## Conclusions

This study of bus drivers at an Australian bus company showed drivers are at increased risk of chronic disease and are impacted by individual, socio-cultural and structural barriers to modifying their health behaviours. In this workplace, strategies could be developed to address these barriers and improve drivers’ health. Ideally, these would use existing workplace resources, be flexible time-wise and focus on reducing drivers’ sitting time (for example, through competitive ‘step challenges’ which publicly acknowledge or reward achievement), facilitating healthier nutritional choices (such as by helping drivers to budget for and prepare or purchase healthy, low-cost meals) and assisting workplace management to develop a culture which supports driver wellness and encourages driver engagement. Targeted health messages using existing workplace digital or mobile technologies may play an important role in future health promotion strategies at this workplace.

This study may provide the basis for further research in other Australian bus companies to identify factors impacting on the health of the larger bus-driving workforce, and the context in which these exist. Such information could inform broader strategies and policies to improve the health of this high-risk but often-overlooked group.

## Supporting information

S1 Appendix Discussion group/interview questions. (DOCX)
