# Supplementary material for: Australian bus drivers’ modifiable and contextual risk factors for chronic disease: A workplace study
Source: PLoS One. 2021 Jul 29;16(7):e0255225. doi: 10.1371/journal.pone.0255225 (PMC8321218; doi:10.1371/journal.pone.0255225)
Supplement: S1 Appendix — (DOCX) [file pone.0255225.s001.docx]

# **S1:** **Appendix**. Discussion Group/Interview Questions

Questions

1. **Can you tell me about your job?**

Prompts

- - What kind of trips do you make?
  - What shifts do you work?

1. **Can you describe how your workplace supports you to be safe and healthy?**

Prompts

- - Are you involved in policy or procedure development?
  - How do you find out about new policies or changes to policies and procedures?
  - How is health and safety information described at induction?

1. **Tell me about your understanding of physical activity?**

Prompts

- - What are the barriers to you being more physically active?
  - What are some ideas you have to change this? Can you describe how you think this issue can be addressed?
  - Can you describe how the workplace can support you to be more physically active?

1. **Tell me about your understanding of healthy eating?**

Prompts

- - What are the barriers to eating healthy foods?
  - What are some ideas you have to change this? Can you describe how you think this issue can be addressed?
  - Can you describe how the workplace can support you?
    1. Facilities in vehicle
    2. Access to food?
    3. Provide information about nutrition of foods

1. **Tell me about the use of digital technologies (e.g. apps, social media, in-house communication systems) in your workplace?**

Prompts

- What types of technologies do you use in your workplace, or in the course of your day-to-day work?
  - How do you use these technologies (e.g. for what purpose)?
  - How often do you use these technologies?

Prompts

- What types of technologies might you use to access information about healthy eating and physical activity?
  - Why do you think you would use these types of technologies?
  - In what ways do you think you would use these types of technologies?
  - Do you think using these technologies may change your health behaviour (e.g. willingness to eat healthily / engage in physical activity? Why?)
  - What might motivate you to use these types of technologies to access health information? (PROMPT: rewards?, challenges?, interactive features?, etc.)
- What are the barriers to the use of digital technologies in your workplace?
- What are some ideas you have to change this? Can you describe how you think this issue can be addressed?
